# Effects of dietary nutrients of the gut microbiota in the long‐tailed dwarf hamster (*Cricetulus longicaudatus*)

**DOI:** 10.1002/ece3.11507

**Published:** 2024-06-25

**Authors:** Kanglin Cao, Mengfan Tao, Xinsheng Pu, Yu Hou, Yue Ren, Wei Liu, Xin'gen Yang

**Affiliations:** ^1^ Shanxi Key Laboratory of Integrated Pest Management in Agriculture, College of Plant Protection Shanxi Agricultural University Taiyuan China; ^2^ Shanxi Forestry and Grassland General Engineering Station Taiyuan China

**Keywords:** 16S rDNA, *Cricetulus longicaudatus*, gut microbiota, metabolic abundance, various nutrient

## Abstract

Gut microbiota is a key factor in maintaining the dietary and metabolic homeostasis of small mammals. To explore the effect of diet on the gut microbiota of the long‐tailed dwarf hamster (*Cricetulus longicaudatus*), 16S rDNA high‐throughput sequencing combined with bioinformatics analysis was used to investigate the succession process of the gut microbiota and effects of different nutrients on the composition and function of the gut microbiota. The results showed that diet structure can significantly influence the composition and function of the gut microbiota, as well as the health of animals. The highest relative abundance of Firmicutes, and the simplest co‐occurrence network occurred in the wild. Whereas the relative abundance of Bacteroidetes is higher and the most complex network structure was observed after 35 days of same feeding. Compared to the other four groups, the relative abundance of Firmicutes in the wheat + peanuts (WP) group was the highest after 35 days of different feeding, and the highest relative abundance of Bacteroidetes occurred in the wheat‐only (WH) group. Bacteroidetes exhibit carbohydrate degradation activity, and Firmicutes are strongly associated with fat uptake. We also found a significant positive correlation between *Lactobacillus* and body weight, indicating that *Lactobacillus* plays a crucial role in modulating fat intake and weight management. This study provides empirical evidence to facilitate the understanding of the co‐evolutionary dynamics between *C. longicaudatus* and their gut microbiota and establishes a theoretical foundation for utilizing gut microbiota in rodent control.

## Graphical abstract

The dietary structure can significantly impact the diversity and community composition of gut microbiota in *Cricetulus longicaudatus*, which primarily inhabits dryland farming areas. The abundance of specific microbiota is closely correlated with the nutrient content of the diet. Gut microbiota plays a pivotal role in regulating nutrient assimilation and metabolic homeostasis in animals.

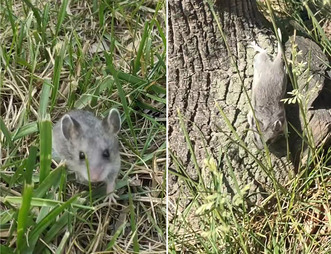

## 1 INTRODUCTION

Mammalian gut microbes colonize the body from birth and gradually form a relatively stable microbial community and a close symbiotic relationship with the host when it matures (Bäckhed et al., 2004; Hooper et al., 2002). These millions of microorganisms not only impact nutrient digestion and absorption but also regulate diverse biological processes encompassing host metabolic functions and environmental adaptability (Liu et al., 2021; Round & Mazmanian, 2009). For instance, the gut microbiota can influence the immune function of mammals, enhancing their resistance to external diseases, promoting tissue generation and repair, and maintaining the host's physiological health (Liu et al., 2012; Nayak, 2010). Moreover, the gut microbiota enhances the host's ability to digest and absorb high‐fiber, low‐protein plants, providing essential nutrients for normal physiological activities and increasing their tolerance to extreme environments (Kohl et al., 2017; Montllor et al., 2002; Tsuchida et al., 2004). The composition and function of the intestinal microbiota are primarily influenced by environmental and genetic factors, with diet presumably being the most prominent environmental factor that shapes the mammalian gut microbial community (Rinninella et al., 2019; Wastyk et al., 2021). In laboratory mice subjected to periodical dietary switching, the relative abundance of Bacteroidales and Clostridiales changed dramatically after just a single day on a new diet (Carmody et al., 2015). Under conditions of natural food feeding in their native habitat, white‐throated woodrats (*Neotoma albigula*) exhibited a significant slowdown in the changes of their gut microbiota, preserving 90% of their original microbial community. However, when fed with artificial diets, the loss rate of their original microbial community reached 38% (Martínez‐Mota et al., 2020). In the wild, the wood mouse (*Apodemus sylvaticus*) alters its dietary preferences (transition between insectivorous and herbivory) based on the seasons and food supply. The relative abundances of *Lactobacillus*, *Helicobacter*, and *Alistipes* in their gut microbiota change significantly (Maurice et al., 2015). Carbohydrates, proteins, and fats are the three major macronutrients that serve as energy sources. They differ greatly in digestibility, and therefore, serve as a completely different nutrient substrate for bacterial metabolism (Zhang et al., 2022). The relative abundance of Firmicutes in fecal samples of mice (*Mus musculus*) raised on a long‐term high‐sugar and high‐fat diet was significantly increased (Parks et al., 2013). While the intake of foods high in saturated fatty acids promoted the proliferation of sulfite‐reducing pathobiont and *Bilophila wadsworthia* (Devkota et al., 2012). Furthermore, the homeostasis of gut microbiota is also influenced by genetic factors. Previous studies have indicated that vertical transmission from the mother and close relatives plays a role in establishing the original microbiota (Moeller et al., 2018; Wang et al., 2020). However, this transmission is not complete and can be influenced by host autonomous selection (Bruijning et al., 2022). Investigations on small mammals such as *Apodemus*, *Microtus*, and *Sorex* have revealed that species genetics overrode the environment in shaping the gut microbial community (Knowles et al., 2019). Nevertheless, recent research suggests that environmental factors have a more substantial impact on gut microbiome composition compared to host genetics, with the gut microbiota being highly responsive to environmental disturbances and exhibiting faster changes than the host genome (Rothschild et al., 2018; Zhang et al., 2023). In both humans and *Mus musculus domesticus*, cecal microbial diversity is predominantly shaped by diet rather than genetic relationships between individuals (Linnenbrink et al., 2013; Rothschild et al., 2018). Notably, convergence of the gut microbiota has been observed in Plateau pika (*Ochotona curzoniae*) and yak (*Bos grunniens*), which further supports horizontal transmission between sympatonic species due to pika fecal‐eating behavior (Fu et al., 2021). Therefore, diet emerges as a pivotal factor driving the convergence of gut microbiota over short‐term fluctuations as well as evolutionary timescales.

Previous studies on wild rodent gut microbiota have mostly targeted rodents living in forests, grasslands, or underground, where food sources are relatively simple, while few studies have focused on rodent gut microbiota in agricultural areas where food structure is more complex. The long‐tailed dwarf hamster (*Cricetulus longicaudatus*) (Rodentia: Cricetidae), one of the main harmful rodents in the dryland farming areas of North China, is mainly distributed in eastern and central China, western and central Mongolia, Tuva, Trans‐Baikal region, and southern Buryatia (Poplavskaya et al., 2018; Yang, Wang, Guo, et al., 2022). Survey results indicated that the long‐tailed dwarf hamster, primarily consuming crop seeds and insects, dominates the rat species in the wild environments of North China, They account for over 70% of the total rodent population in many regions. The high population density can lead to a reduction in crop yields by 5%–15%, it poses a significant threat to the healthy development of agriculture in the region (Yang, Wang, Zou, et al., 2022). Our previous studies have indicated that this species in Shanxi Province exhibits above‐average genetic diversity (Yang, Wang, Guo, et al., 2022). However, existing scientific literature provides evidence that environment‐induced variations in gut microbiota determine host phenotypes and fitness, despite evidence that host gut microbial diversity is the consequence of genetic variations (Zhang et al., 2023). Regrettably, the investigation into the diversity of the gut microbiota in the long‐tailed dwarf hamster remains largely unexplored. Mounting evidence suggests that the diversity and biological characteristics of rodent gut microbiota hold significant potential for application in controlling rodents (Feng et al., 2020; Schubert et al., 2015; Sekirov et al., 2008).

It is not clear how food, a key factor that affects the gut microbiota of the long‐tailed dwarf hamster, causes changes in the structure of the gut microbial community. In order to clarify the gut microbial composition of *C. longicaudatus* under different ecological environments and the effects of artificial feeding on their gut microbial composition. This study analyzed the species composition and diversity of the gut microbiota in the long‐tailed dwarf hamster under both wild and artificial feeding conditions using 16S rDNA high‐throughput sequencing. Additionally, we explored the interaction of different nutrients and specific gut microbiota and predicted the biological function of the gut microbiota. To provide theoretical support for a better understanding of the ecological adaptability of the long‐tailed dwarf hamster to different environments and to elucidate the co‐evolution of small mammals and gut microbiota.

## 2 MATERIALS AND METHODS

### 2.1 *Cricetulus longicaudatus* collection

**FIGURE 1:**
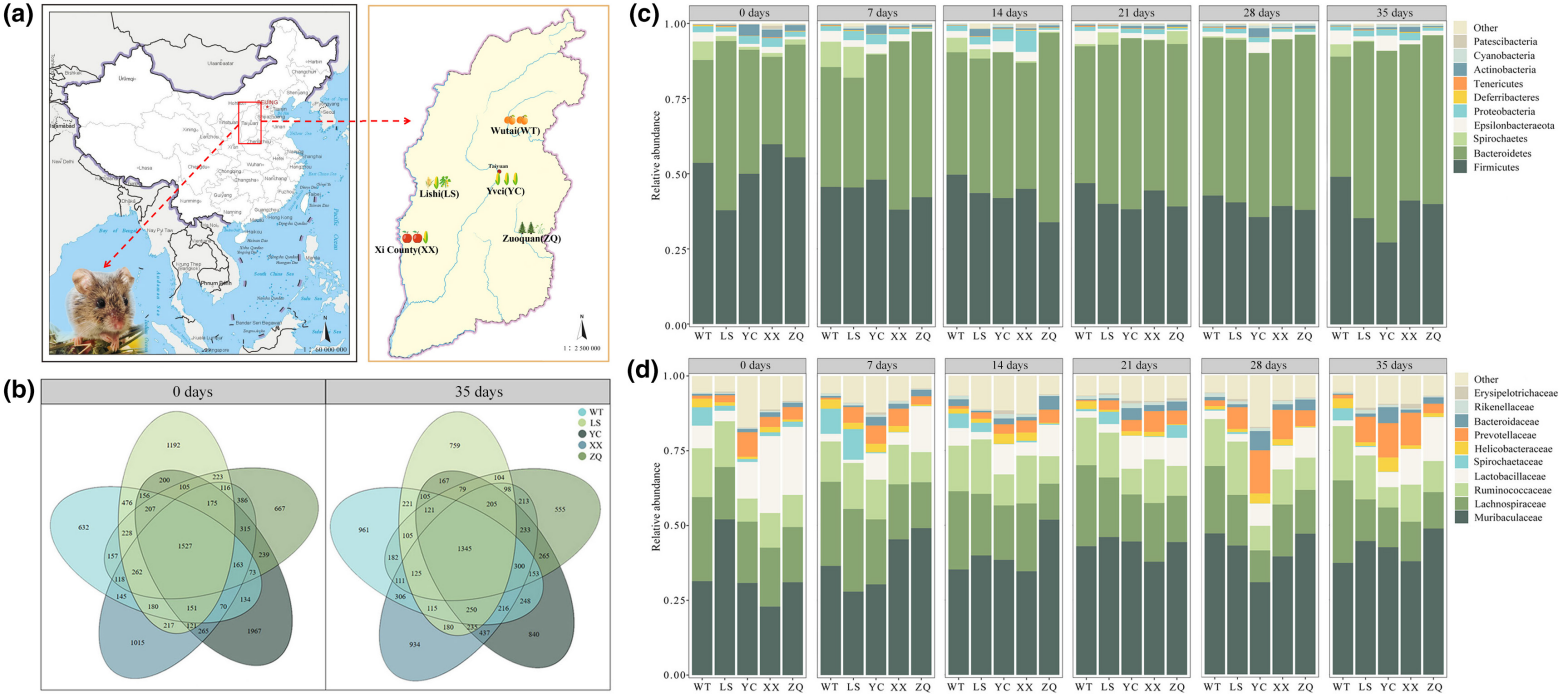
Sample collection and distribution of gut microbiota under same feeding in *Cricetulus longicaudatus*. (a) The sampling sites and photo of *C. longicaudatus* (The maps are from http://bzdt.ch.mnr.gov.cn/, and the photo of *C. longicaudatus* from authors'own); (b) OTU Venn diagram of gut microbiota; (c) Distribution of gut microbiota at the phylum level under same feeding; (d) Distribution of gut microbiota at the family level under same feeding.

The sampling time was from July 2022 to September 2022. The wild healthy adult males of *C. longicaudatus* were trapped using live trapping and then locked in cages previously sterilized with 75% alcohol. This was performed in Wutai County (WT, *n* = 5), Lishi District (LS, *n* = 5), Yuci District (YC, *n* = 5), Xi County (XX, *n* = 5), and Zuoquan County (ZQ, *n* = 5) in Shanxi, China (Figure 1a; Table 1). The elevation of the five regions ranges from 900 to 1200 m, while the mean summer temperature varies between 18°C and 23°C. WT and ZQ are situated in the rocky mountain area, characterized by apricot as the dominant vegetation in WT and Chinese pine along with feather fingergrass as the prevailing vegetation in ZQ. LS, YC, and XX are located in the loess hilly region. In LS, soybean, millet, and maize constitute the dominant vegetation. Maize is predominantly found in YC, whereas apple and maize dominate XX. The animals were immediately weighed and transported back to the laboratory. *C. longicaudatus* were provided ad libitum access to water and exposed to natural light. They were individually housed in cages (33 cm × 21.5 cm × 16 cm) under 20°C–25°C conditions.

**TABLE 1:** Geographical locations and main conditions of *Cricetulus longicaudatus*.

Sample sites	Wutai, Xinzhou	Lishi, Lvliang	Yvci, Jinzhong	Xi County, Linfeng	Zuoquan, Jinzhong
Groups	WT	LS	YC	XX	ZQ
Sample number	5	5	5	5	5
Geographic coordinates	113°27′ E 38°46′ N	111°09′ E 37°31′ N	112°50′ E 37°40′ N	110°50′ E 36°38′ N	113°17′ E 37°05′ N
Altitude/m	936	1175	896	1020	1089
Landscape	Rocky Mountain Area	Loess Hilly Area	Loess Hilly Area	Loess Hilly Area	Rocky Mountain Area
Summer average temperature/°C	18.5	22.5	21.5	21.5	20.2
Site type	Orchard	Farmland	Farmland	Orchard	Young forest land
Dominant vegetation	Apricot	Soybean, millet, and maize	Maize	Apple and maize	Chinese pine and feather fingergrass

All procedures were conducted according to the Regulations for the Administration of Laboratory Animals established by the Ministry of Science and Technology of the People's Republic of China (2017 Revision).

#### 2.1.1 Fecal sample collection

The experiment was divided into two phases. Fresh feces were collected in 2 mL tubes (Sigma‐Aldrich, St. Louis, MO, USA), denoted as day 0 of the first phase. Fecal samples were collected immediately in the wild and stored at −20°C and then were transferred to a −80°C ultra‐low temperature refrigerator for storage. Following, the animals were grouped geographically indoors. Aimed at ensuring nutritional balance and simulating food sources of the wild, we abundantly provided with a diet consisting of wheat, apples, peanuts, and mealworms (all of them are sourced from reputable supermarkets), with each component weighing more than 10 g (Table 2). Fecal samples from captives were collected and recorded body weight of each individual on days 7, 14, 21, 28, and 35, respectively. In the second phase, 25 individuals were rerandomized into five groups, denoted as day 0. Each group consisted of five individuals and was fed one of the following diets: wheat only (WH), wheat + apples (WA), wheat + peanuts (WP), wheat + mealworms (WM), and wheat + apples + peanuts + mealworms (MIX). Fecal samples were collected and recorded body weight at 7, 14, 21, 28, and 35 days. All fecal samples were stored at −80°C until DNA extractions were performed.

**TABLE 2:** Food nutrition contents.

Name	Carbohydrate/%	Dietary fiber%	Fat/%	Protein/%
Wheat	64.4	10.8	1.3	11.9
Apple	12.3	1.2	0.2	0.2
Peanut	16.2	5.5	44.3	24.8
Mealworm	10.0	2.8	34.1	47.4

*Note*: Ye et al., 1997; https://yingyang.911cha.com/.

#### 2.1.2 Fecal DNA extraction and sequencing

The total DNA of fecal microbial communities was extracted using a magnetic bead‐based soil and fecal genomic DNA extraction kit, with primers 341F (5′‐CCTAYGGGRBGCASCAG‐3′) and 806R (5′‐GGACTACNNGGGTATCTAAT‐3′). The PCR reaction system was as follows: Phusion Master Mix (2×) 15 μL, Primer (2 μM) 3 μL, gDNA (1 ng/μL) 10 μL, with ddH_2_O filling up to 30 μL. PCR reaction procedure: pre‐denaturation at 98°C for 1 min; denaturation at 98°C for 10 s, annealing at 50°C for 30 s, extension at 72°C for 30 s, for a total of 30 cycles; final extension at 72°C for 5 min. An equal volume of 1× loading buffer was mixed with the PCR product, and electrophoresis detection was performed on a 2% agarose gel, selecting bands of 400–450 bp for recovery. The library was constructed using the TruSeq® DNA PCR‐Free Sample Preparation Kit. Quality control was carried out with the Qubit@2.0 Fluorometer and Agilent 2100 Bioanalyzer system. After the library passed the quality check, fecal sample 16S rDNA sequencing was completed on the Illumina NovaSeq 6000 platform by the Shanghai Zhongke Xinsheng Life Biotechnology Co., Ltd.

#### 2.1.3 16S rDNA data processing

Fastp 0.19.6 software was used for quality control and raw data filtering (Chen et al., 2018). Paired‐end sequences based on overlapping regions were merged in Flash 1.2.11 software (Magoč & Salzberg, 2011). Filtering the obtained merged sequences, we retained sequences with length > 200 bp, without ambiguous bases, and with an average sequence quality score ≥ 20. Detected chimeric sequences were removed using Uchime 11.0 to obtain high‐quality, valid data. The valid data for clustering analysis using the Uparse algorithm were divided into operational taxonomic units (OTUs) based on a similarity threshold of ≥97% (Edgar, 2013), and the data were standardized to a minimum sample sequence count of 41,737. The standardized process was executed by single_rarefaction.py, followed by OTU clustering analysis after distinguishing sequences by sample. The representative sequences of OTUs were classified using RDP classifier 2.11, and the composition of each sample's community was tallied. The comparison was done against the Silva 132 database (http://www.arb‐silva.de) with a confidence threshold of 70%, and the composition of community species in different taxonomic levels was tabulated for each sample (Wang et al., 2007).

#### 2.1.4 Statistical analysis

Microbial diversity analysis was performed on the APT‐BioCloud cloud data analysis platform. Venn diagrams were used to show common and unique OTUs among different groups. The diversity of the gut microbiota in the five populations was assessed using QIIME software, with analysis based on the Shannon index. Principal component analysis was performed on the gut microbiota communities, and intergroup beta diversity distance was calculated using unweighted UniFrac algorithms. Anosim analysis was used to check whether the dissimilarities between the groups were significantly greater than the dissimilarities within the groups. OTUs were distributed in different time nodes and the top 200 of them were chosen for Spearman's correlation analysis. It was generated by screening out significant correlations (FDR *p* < .05) with an absolute coefficient value greater than 0.6 from the results and then drawing the co‐occurrence networks using Gephi v0.9.2 software (Jacomy et al., 2014). The structural differences in the gut microbiota among the five groups were compared using LefSe analysis (Segata et al., 2011). The LDA values of each species were calculated using linear discriminant analysis to estimate their impact on the differential effect and identify species exhibiting significant intergroup differences in abundance changes. If the species had a log_10_ (LDA) value greater than 3 and a *p*‐value less than .05, it was considered significant among groups. Heatmaps were plotted using the R package “pheatmap.” KEGG functional prediction based on 16S rDNA amplicon sequencing results was performed using PICRUSt 2 software. All experimental results were expressed as mean values ± standard deviation (mean ± SD). The relative abundance and alpha diversity of multigroup samples were calculated using SPSS 26.0 software through repeated measures ANOVA, while Duncan's new multiple range test was employed to assess the significance of metabolite abundance differences between groups. Weight differences were analyzed using a paired sample *t*‐test. Statistical differences between treatments were considered significant at *p < .05, **p < .01, and ***p < .001.

## 3 RESULTS

### 3.1 Species annotation and evaluation of the gut bacteria in *Cricetulus longicaudatus*

We obtained 24,597,786 effective sequences from 275 fecal samples of *C. longicaudatus* through 16S rDNA sequencing. Using a 97% similarity threshold for clustering, a total of 19,439 OTUs were detected, which were classified into 47 phyla, 106 classes, 240 orders, 441 families, 1032 genera, and 668 species.

**FIGURE 2:**
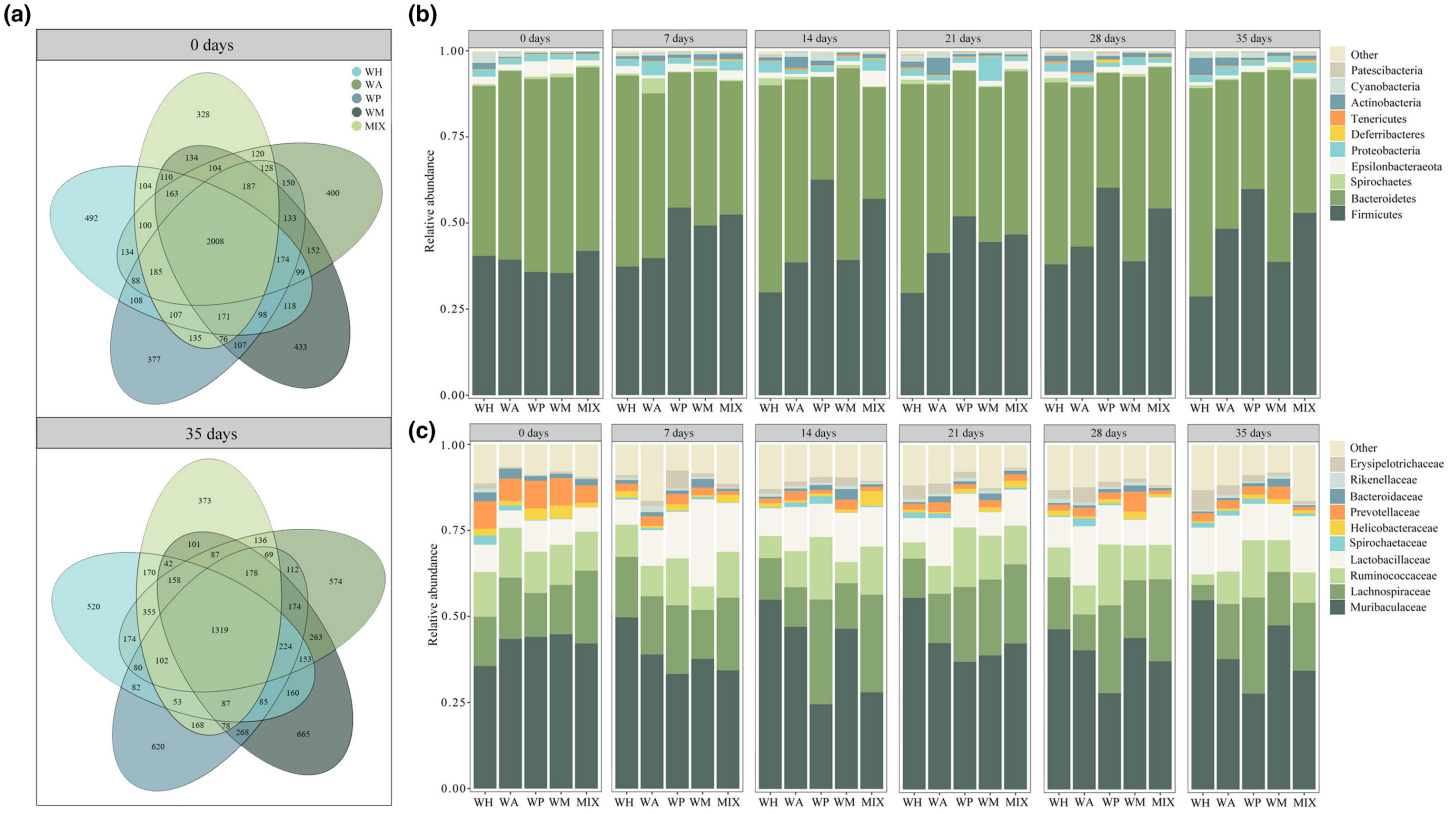
Distribution of gut microbiota under different feeding in *Cricetulus longicaudatus*. (a) OTU Venn diagram of gut microbiota; (b) Phylum level; (c) Family level.

In the wild (day 0 of the first phase), the Venn diagram showed that the number of OTUs shared by WT, LS, YC, XX, and ZQ was 1527, accounting for 12.85% of the total OTUs (Figure 1b). After 35 days of same feeding, the shared number of OTUs among the five populations was 1345, but the proportion increased to 13.23% (Figure 1b). After randomization into the second phase of the study, the number of OTUs shared by WH, WA, WP, WM, and MIX decreased from 2008 (0 day) to 1319 (35 days), and their proportion in the total OTUs decreased from 27.80% (0 day) to 17.29% (35 days) (Figure 2a).

### 3.2 Gut microbial taxonomic difference among groups

At the phylum level, the common dominant phyla of the gut microbiota of *C. longicaudatus* in the wild were Firmicute and Bacteroidetes (0 day in Figure 1c). The relative abundance of Firmicutes in the LS group was significantly lower than that in WT, XX, and ZQ groups (*p* < .05) (0 day in Figure 1c). The relative abundance of Bacteroidetes in LS was significantly higher than that in WT, XX, and ZQ (*p* < .05) (0 day in Figure 1c). While there were no significant differences in the relative abundances of the two phyla in YC compared to other groups (*p* > .05) (0 day in Figure 1c). At the family level, the major families were Muribaculaceae, Lachnospiraceae, Lactobacillaceae, and Ruminococcaceae. The relative abundance of Muribaculaceae was the highest among the five groups, and its relative abundance in LS was significantly higher than that in the other four groups (*p* < .05) (0 day in Figure 1d). The relative abundance of Lactobacillaceae was significantly higher in XX compared to WT and LS (*p* < .05) (0 day in Figure 1d). Furthermore, the relative abundances of Ruminococcaceae were significantly higher in WT and LS than in YC (*p* < .05) (0 day in Figure 1d). Additionally, the highest relative abundances of Spirochaetaceae and Prevotellaceae occurred in WT and YC, respectively (0 day in Figure 1d).

### 3.3 Analysis of the gut microbiota under same feeding

At the phylum level, the relative abundances of Firmicutes (population: *F*_4,20_ = 3.04, *p* = .041; time: *F*_5,100_ = 4.46, *p* = .001; population×time: *F*_20,100_ = 1.15, *p* = .313) and Bacteroidetes (population: *F*_4,20_ = 3.70, *p* = .021; time: *F*_5,100_ = 5.28, *p* < .001; population×time: *F*_20,100_ = 1.57, *p* = .075) in the gut microbiota of long‐tailed dwarf hamsters were significantly influenced by both time and population. During the same feeding process (day 0 to 35 of the first phase), an increase in the relative abundance of Bacteroidetes was observed in the five groups, while a decrease in the relative abundance of Firmicutes was noted (Figure 1c). Among them, the relative abundances of these two phyla exhibited significant changes on day 35 in YC and XX compared to them in the wild (*p* < .05) (Figure 1c). At 35 days, the relative abundance of Bacteroidetes and Firmicutes was not significantly different among the LS, YC, XX, and ZQ (*p* > .05) (35 days in Figure 1c). At the family level, the relative abundance of Lactobacillaceae (population: *F*_4,20_ = 12.46, *p* < .001; time: *F*_5,16_ = 1.73, *p* = .185; population×time: *F*_20,76_ = 1.68, *p* = .056) in the gut microbiota was significantly influenced by population. The relative abundances of Muribaculaceae in the five groups were not significantly different at 35 days, and there were no significant differences in the relative abundances of Lactobacillaceae among WT, LS, and YC (*p* > .05) (35 days in Figure 1d).

### 3.4 Effects of various dietary patterns on gut microbiota

After 35 days of same feeding, the hamsters were randomly allocated into five groups (*n* = 5), marking the commencement of day 0 for the second phase of the study. The relative abundances of the dominant phyla and families were no significant differences among the five groups (*p* > .05) (0 day in Figure 2b,c).

After 35 days of different feeding, at the phylum level, the grouping significantly influenced the relative abundance of Firmicutes (group: *F*_4,20_ = 9.044, *p* < .001; time: *F*_5,100_ = 1.71, *p* = .140; group×time: *F*_20,100_ = 1.26, *p* = .223) and Bacteroidetes (group: *F*_4,20_ = 7.58, *p* < .001; time: *F*_5,100_ = 1.86, *p* = .109; group×time: *F*_20,100_ = 1.31, *p* = .194) in the gut microbiota. The relative abundance of Firmicutes in the WP group showed a significant increase at the phylum level (*p* < .01), while there was a significant decrease in the relative abundance of Bacteroidetes (*p* < .01) (Figure 2b). The relative abundance of Firmicutes in the WH group was significantly lower than that in the WA, WP, and MIX groups (*p* < .05) (35 days in Figure 2b). However, the WH group exhibited a significantly higher level of Bacteroidetes compared to the WA, WP, and MIX groups (*p* < .05) (35 days in Figure 2b).

At the family level, the relative abundances of Muribaculaceae (group: *F*_4,20_ = 6.78, *p* = .001; time: *F*_5,100_ = 0.55, *p* = .737; group×time: *F*_20,100_ = 1.47, *p* = .109) and Lachnospiraceae (group: *F*_4,20_ = 9.94, *p* < .001; time: *F*_5,100_ = 0.34, *p* = .887; group×time: *F*_20,100_ = 1.22, *p* = .252) were significantly influenced by group. Grouping and interaction between group and time significantly influenced the relative abundance of Ruminococcaceae (group: *F*_4,20_ = 7.20, *p* < .001; time: *F*_5,16_ = 1.16, *p* = .371; group×time: *F*_20,76_ = 2.49, *p* = .002). The relative abundance of Lactobacillaceae was significantly influenced by time (group: *F*_4,20_ = 0.29, *p* = .883; time: *F*_5,16_ = 3.66, *p* = .021; group×time: *F*_20,76_ = 0.84, *p* = .659). The relative abundance of Muribaculaceae was significantly increased in the WH group compared to day 0 (*p* < .05) (Figure 2c). Conversely, a significant decrease in the relative abundance of Ruminococcaceae was observed (*p* < .01) (Figure 2c). WP group showed a significant increase in the relative abundance of Lachnospiraceae (*p* < .05), while the WA group exhibited a significant increase in the relative abundance of Lactobacillaceae (*p* < .05) (Figure 2c). At 35 days, the relative abundance of Muribaculaceae in the WH group was significantly higher than that in the WA, WP, and MIX groups (*p* < .05). Moreover, the WH group exhibited a significantly lower relative abundance of Lachnospiraceae compared to the WP and MIX groups (*p* < .05). Additionally, the WH group displayed a significantly lower relative abundance of Ruminococcaceae compared to all other four groups (*p* < .05), while the WP group showed a significantly higher relative abundance than all other four groups (*p* < .01) (35 days in Figure 2c).

### 3.5 Diet differences in gut microbiota co‐occurrence networks

**FIGURE 3:**
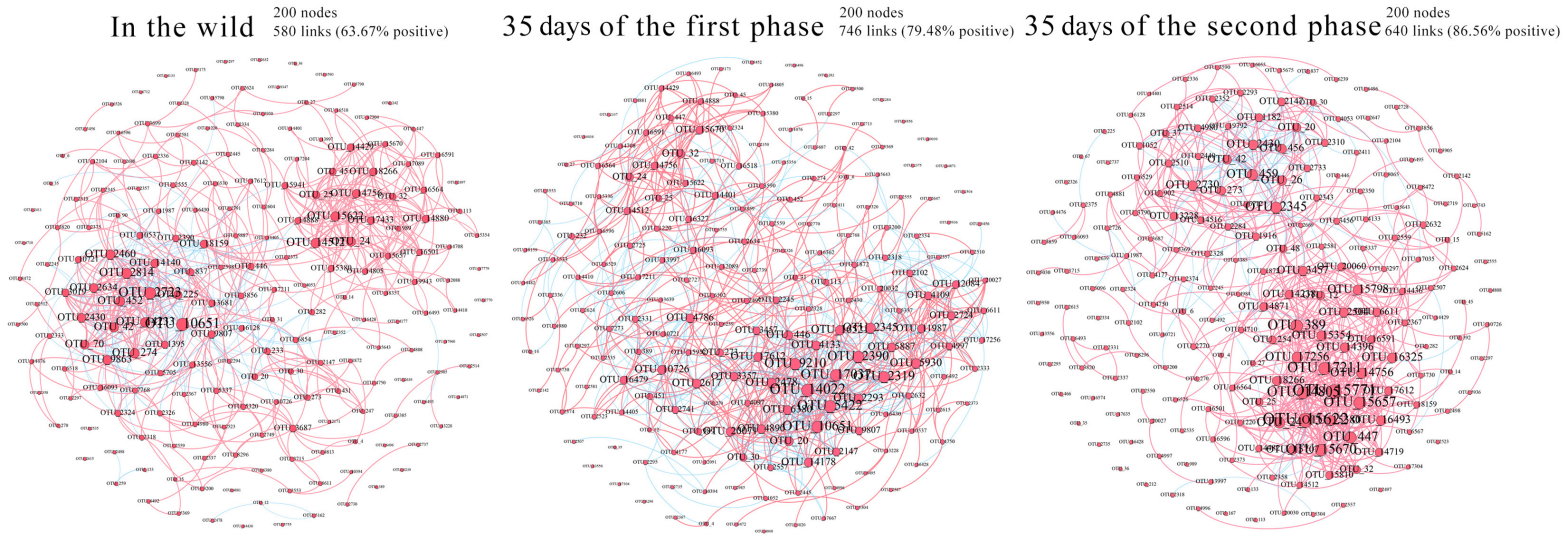
Co‐occurrence networks of the top 200 operational taxonomic units (OTUs) among different time nodes. Nodes represent OTUs and their sizes indicate different relative abundance. Links between the nodes indicate a significant correlation between two OTUs (Spearman's correlation greater than 0.6 or lower than −0.6). Line color reflects direction (blue: negative; pink: positive).

The gut microbiota of *C. longicaudatus* had the most complex network structure with 200 nodes and 746 links of same feeding at 35 days of the first phase, 200 nodes and 580 links in the wild, 200 nodes and 640 links at 35 days of the second phase (Figure 3). Several other important network topological properties such as average degree, average weighted degree, and density also showed differences in the network structures of *C. longicaudatus* (Figure 3). It was worth noting that at 35 days of the first phase and the second phase, the positive correlation in the networks occupied an absolute dominant position (more than 79%), whereas, in the wild, the negative correlation increased (more than 36%) (Figure 3).

### 3.6 Gut microbiota diversity

**FIGURE 4:**
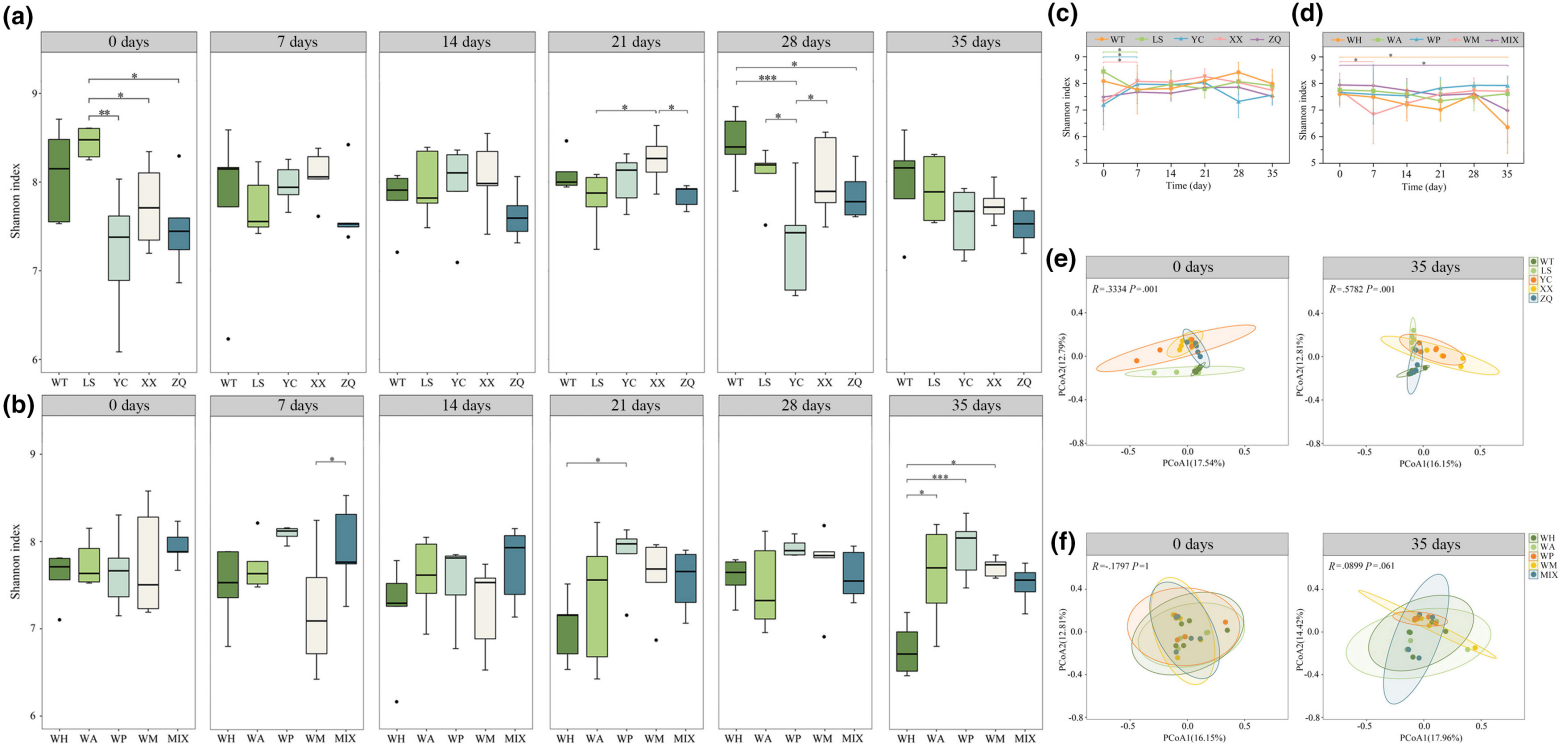
Alpha diversity and Beta diversity of the gut microbiota. (a, c) Shannon index under same feeding; (b, d) Shannon index under different feeding; (e) PCoA based on unweighted UniFrac distances under same feeding; (f) PCoA based on unweighted UniFrac distances under different feeding. Data are mean ± SD. *p < .05, **p < .01, ***p < .001.

Based on the analysis of α and β diversity, the Shannon index of the gut microbiota in the LS group exhibited a significantly higher level compared to that of YC, XX, and ZQ in the wild (*p* < .05) (0 days in Figure 4a). The results of repeated measure analysis of variance showed that both the population and the interaction between population and time significantly influenced the Shannon index of gut microbiota in long‐tailed dwarf hamsters (population: *F*_4,20_ = 3.06, *p* = .04; time: *F*_5,100_ = 1.67, *p* = .149; population×time: *F*_20,100_ = 2.08, *p* = .009). Following the same feeding for 7 days, there was a significant decrease in the Shannon index of LS (*p* < .05), while both YC and XX demonstrated a significant increase (*p* < .05) (Figure 4c).

After 35 days of same feeding, there were no significant differences in the Shannon index of the five groups (*p* > .05) (35 days in Figure 4a). After rerandomization, when *C. longicaudatus* were fed with different foods, there were no significant differences in the Shannon index of the gut microbiota among the WH, WA, WP, WM, and MIX groups (*p* > .05) (0 day in Figure 4b). The results of repeated measure analysis of variance showed that grouping significantly influenced the Shannon index of gut microbiota (group: *F*_4,20_ = 3.13, *p =* .037; time: *F*_5,16_ = 2.30, *p* = .093; group×time: *F*_20,76_ = 1.13, *p* = .342). The Shannon index of the WH group was significantly decreased at 35 days (*p* < .05) (Figure 4d). Following a feeding period of 35 days, the Shannon index of the gut microbiota in the WH group was significantly lower than that in the WA, WP, and WM groups (*p* < .05) (35 days in Figure 4b).

We analyzed the shifts in bacterial communities among wild and same feeding samples using principal coordinate analysis (PCoA). Unweighted UniFrac distance matrices were used as measures of beta diversity. The result showed that there was a significant separation between all five groups in the wild (*R* = .3334, *p* = .001), while the overlap was significant among the groups after 35 days of same feeding, and the structural similarity was high in the gut microbiota (*R* = .5782, *p* = .001) (Figure 4e). After rerandomization and 35 days of various diets, the samples showed a discrete trend among the five groups, however, no statistically significant differences were observed (*R* = .0899, *p* = .061) (Figure 4f).

### 3.7 LEfSe analysis of the gut microbiota

**FIGURE 5:**
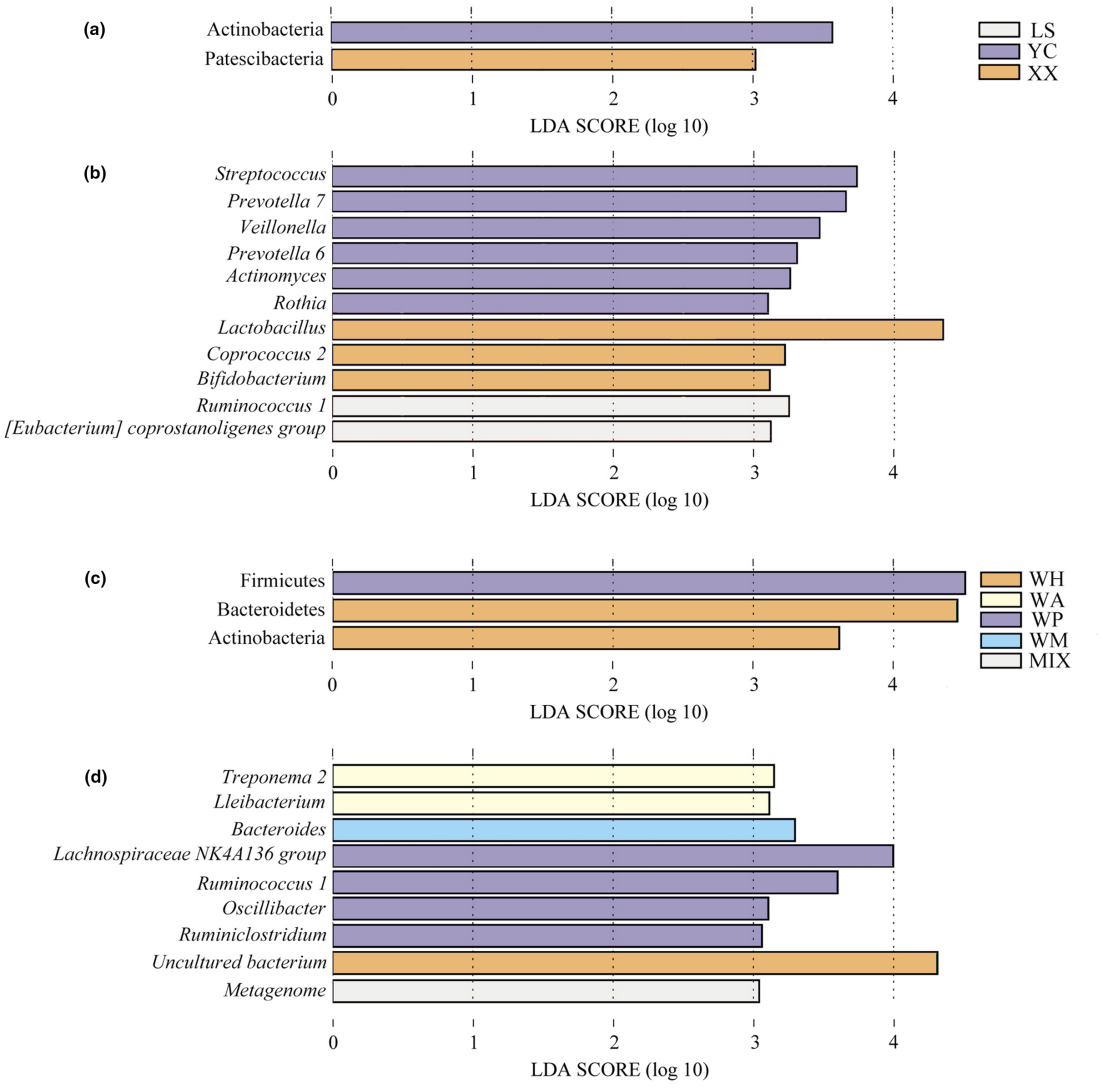
LEfSe analysis of the gut microbiota in *Cricetulus longicaudatus*. (a) Phylum level in the wild; (b) Genus level in the wild; (c) Phylum level after 35 days under different feeding; (d) Genus level after 35 days under different feeding.

The LEfSe analysis indicated that two phyla and 11 genera were enriched differently in the wild (Figure 5a,b). The abundances of Actinobacteria, *Streptococcus*, *Prevotella* 7, *Veillonella*, *Prevotella* 6, *Actinomyces*, and *Rothia* in the gut microbiota of YC were significantly higher compared to these in the other four groups (Figure 5a,b). While the abundances of Patescibacteria, *Lactobacillus*, *Coprococcus* 2, and *Bifidobacterium* in the gut microbiota of XX were significantly higher (Figure 5a,b). After 35 days of different feeding, the five groups exhibited distinct enrichment in terms of three phyla and nine genera (Figure 5c,d). The abundances of Bacteroidetes, Actinobacteria, and Uncultured bacterium in the gut microbiota of WH were significantly higher compared to those in the WA, WP, WM, and MIX groups (Figure 5c,d). The abundances of Firmicutes, Lachnospiraceae *NK4A136 group*, *Ruminococcus* 1, *Oscillibacter*, and *Ruminiclostridium* in the gut microbiota of WP were significantly higher (Figure 5c,d).

### 3.8 Functional predictions of the gut microbiota

**FIGURE 6:**
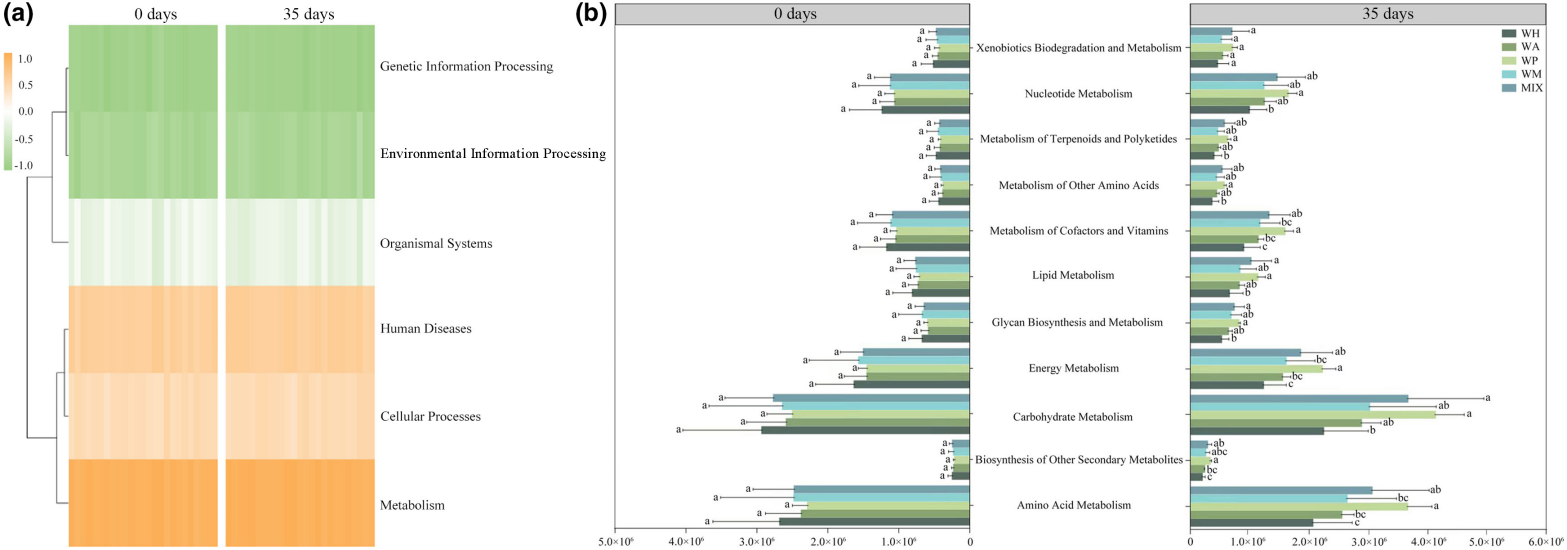
KEGG functional annotation of the gut microbiota in *Cricetulus longicaudatus* under different feeding. (a) The top level; (b) The second level. Data are mean ± SD. Different letters in the same metabolic pathway indicate there is a significant difference between groups (*p* < .05)

The gut microbiota from fecal samples were predicted into three levels based on KEGG functional annotation. At the top level, genetic information processing, environmental information processing, organismal systems, human diseases, cellular processes, and metabolism were six primary categories. The result showed that the abundance of metabolic pathways was always the highest (Figure 6a). At the second level, carbohydrate metabolism, amino acid metabolism, energy metabolism, nucleotide metabolism, and metabolism of cofactors and vitamins were the primary functions (Figure 6b). The metabolic abundance of the gut microbiota in the WP group was the highest among the five groups, while that in the WH group was the lowest (Figure 6b). At the third level, we identified the metabolic pathways with the top six abundance changes in each group. Compared to the other four groups, the metabolic abundance of carbon fixation pathways in prokaryotes (Energy metabolism) was higher in the WH group, while the metabolic abundance of glycolysis/gluconeogenesis (Carbohydrate metabolism) was lower (Table 3).

**TABLE 3:** The third level KEGG functional annotation of the gut microbiota under different feeding.

Term (top 6)	Function	KEGG functional annotation
Compare WH to WA group	WH: Energy metabolism, amino acid metabolism, metabolism of cofactors and vitamins WA: Carbohydrate metabolism, glycan biosynthesis and metabolism	WH: Methane metabolism, arginine and proline metabolism, porphyrin and chlorophyll metabolism, carbon fixation pathways in prokaryotes WA: Glycolysis/gluconeogenesis, Peptidoglycan biosynthesis, galactose metabolism, propanoate metabolism
Compare WH to WP group	WH: Energy metabolism WP: Carbohydrate metabolism	WH: Carbon fixation pathways in prokaryotes WP: Glycolysis/gluconeogenesis
Compare WH to WM group	WH: Energy metabolism, amino acid metabolism, carbohydrate metabolism WM: Carbohydrate metabolism	WH: Methane metabolism, arginine and proline metabolism, pyruvate metabolism, carbon fixation pathways in prokaryotes WM: Starch and sucrose metabolism, Fructose and mannose metabolism, galactose metabolism, glycolysis/gluconeogenesi, pentose phosphate pathway
Compare WH to MIX group	WH: Amino acid metabolism, Metabolism of cofactors and vitamins, energy metabolism MIX: Carbohydrate metabolism	WH: Arginine and proline metabolism, Porphyrin and chlorophyll metabolism, Carbon fixation pathways in prokaryotes MIX: Glycolysis/Gluconeogenesis, fructose and mannose metabolism, starch and sucrose metabolism

Abbreviations: MIX, wheat + apples + peanuts + mealworms; WA, wheat + apples; WH, wheat only; WM, wheat + mealworms; WP, wheat + peanuts.

### 3.9 Correlation analysis between the gut microbiota and body weight

**FIGURE 7:**
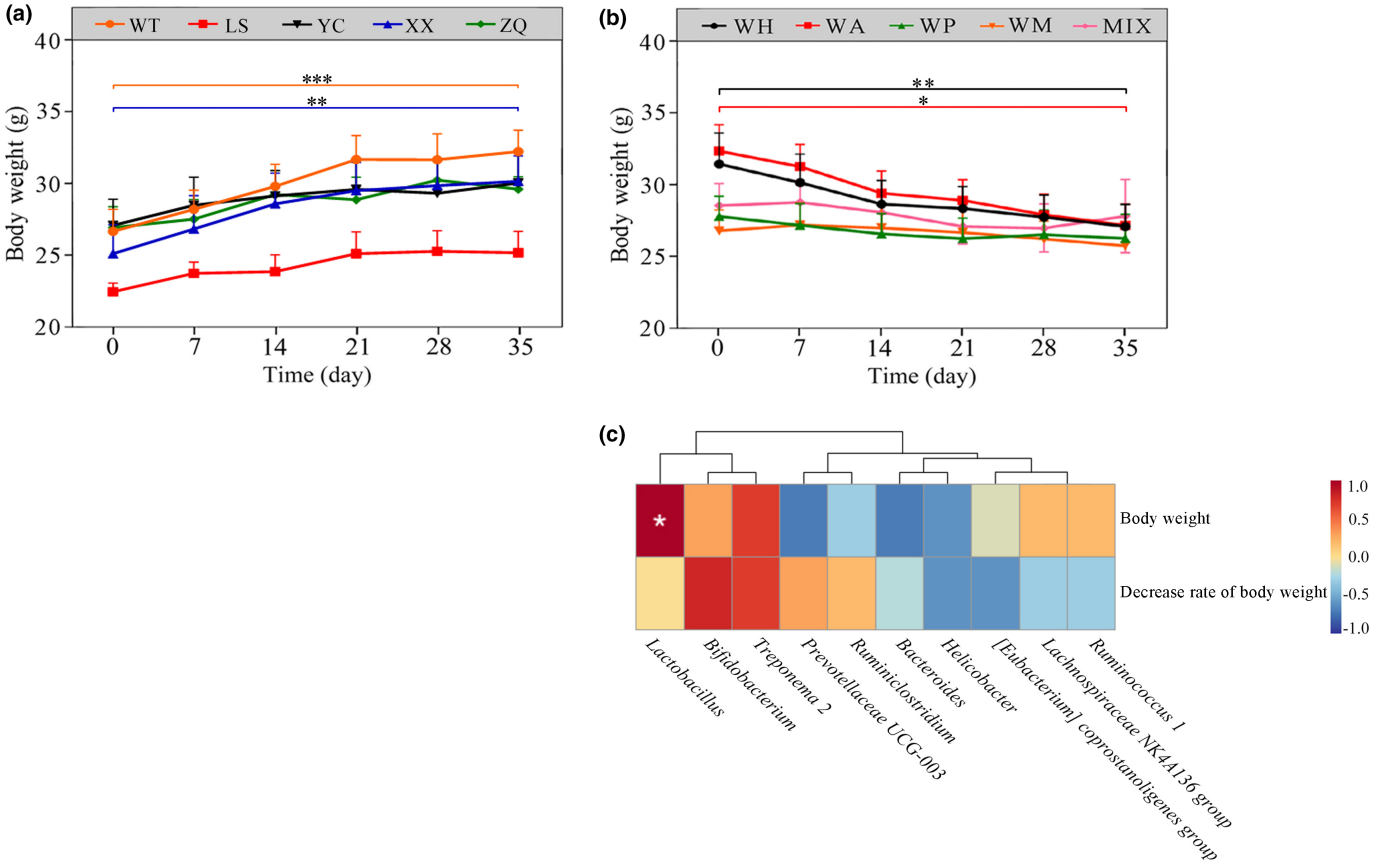
(a) Changes in body weight in five groups under same feeding; (b) Changes in body weight in five groups under different feeding; (c) Correlation analysis between gut microbiota (genus level) and body weight, decrease rate of body weight under different feeding. Data are mean ± SD. *p < .05, **p < .01, ***p < .001.

After 35 days of same diet, the body weight of long‐tailed dwarf hamsters exhibited a noticeable upward trend in comparison to them in the wild, with the WT (*t* = −14.28, *p* < .001) and XX (*t* = −4.11, *p* = .015) populations exhibited a significant increase in body weight (Figure 7a).

After 35 days of different feeding, the body weight of the WH, WA, WP, and WM groups showed a decreasing trend, and the decrease was significant in WH (*t* = 6.24, *p* = .003) and WA (*t* = 3.33, *p* = .029) groups (Figure 7b). The correlation between body weight and the dominant genera (top 10 in the relative abundance of samples) in the feces showed that body weight was significantly positively correlated with the abundance of *Lactobacillus* (*p* < .05) (Figure 7c).

## 4 DISCUSSION

The gut microbiota plays a crucial role in the host's nutrient metabolism (Fan et al., 2022; Linnenbrink et al., 2013). In order to ensure nutritional balance for *C. longicaudatus*, and closely simulate the wild diet, which consists of complex food sources in their natural environment, we investigated the dynamic changes in *C. longicaudatus* during both consistent and varied feeding processes and predicted the metabolic function of them. This study not only unveils the significant contribution of the gut microbiota to host ecological adaptation but also establishes a theoretical foundation for utilizing gut microbiota in rodent control.

The composition of the gut microbiota in wild and captive *C. longicaudatus* was analyzed, along with the changes in the gut microbiota resulting from the same and different feeding practices. The results revealed that Firmicutes and Bacteroidetes were predominant in the gut microbiota of both wild and captive *C. longicaudatus*. In fecal samples from herbivores, the proportions of Firmicutes and Bacteroidetes can account for more than 90% (Guan et al., 2017; Ishaq & Wright, 2014). In this study, Firmicutes and Bacteroidetes constituted more than 80% of the gut microbiota in *C. longicaudatus*. This may indicate that in the wild, the species mainly feeds on plant‐derived food such as crops and grass seeds. Our study also revealed that the gut microbiota of wild *C. longicaudatus* were predominantly composed of Firmicutes, whereas Bacteroides dominated the gut microbiota of the captive. This finding aligns with previous research on other wild animals (Fernando et al., 2010; Li et al., 2015). Firmicutes can not only convert cellulose into volatile fatty acids for use by the host but also improve the digestion and absorption of plant nutrients in the host gut and enhance host autoimmunity to prevent pathogen interference. This reflects the co‐evolution between wild animals and gut microbiota (Fernando et al., 2010; Guan et al., 2017). After transitioning to captivity, most groups of Bacteroidetes were sensitive to changes in food nutrients due to their functions in degrading carbohydrates and proteins, gradually dominating the gut microbiota within a certain period of time (Becker et al., 2014; Flint et al., 2012).

The diversity of gut microbiota in rodents can be attributed directly to variations in their diet (Turnbaugh et al., 2009; Wu et al., 2011). In the wild, the diversity of gut microbiota LS group was found to be higher compared to other groups, primarily attributed to the intricate agricultural planting structure in Lishi and the abundant food availability for *C. longicaudatus* in their natural habitat. Conversely, when kept in captivity, there was a significant reduction observed in gut microbiota diversity, indicating that wild groups are exposed to a more diverse microbiome through environmental sources (such as a wider range of habitats, seasonal variations, social interactions, and varied diet), which contributes to an increased richness of gut microbiota (Adair & Douglas, 2017; Bobbie et al., 2017; Maurice et al., 2015). However, *C. longicaudatus* encounter food shortages and predator threats in their natural habitat. It has been confirmed that environmental stress is inversely related to positive network cohesion and complexity (Hernandez et al., 2021). Consequently, the co‐occurrence network of gut microbiota in captive long‐tailed dwarf hamsters exhibits greater complexity compared to their counterparts in the wild (Figure 3).

The acquisition of sufficient nutrients from food poses a fundamental challenge for wildlife species. Previous research has demonstrated that the gut microbiota and its metabolites not only govern mammalian health but also play a crucial role in facilitating animals' adaptation to drastic dietary alterations and energy constraints (Schmidt et al., 2019; Turnbaugh et al., 2006). In this study, the relative abundance of major gut microbiota in *C. longicaudatus* of different regions gradually converged under same feeding conditions, however, short‐term intake of various nutrients resulted in significant differences in the gut microbiota of *C. longicaudatus*, indicating that the gut microbiota play a role in regulating nutrient intake for this species. The results of this study indicated that starch, as the primary energy source for the WH group, could lead to an increase in the relative abundance of Bacteroidetes in their gut. Certain bacteria belonging to the Bacteroides possess a significant carbohydrate degradation activity reservoir, capable of fermenting various plant polysaccharides to provide energy for the host's life activities (Comstock & Coyne, 2003; Kaoutari et al., 2013). Consuming peanuts can significantly increase the abundance of Firmicutes in the gut microbiota and enrich the expression of genes involved in short‐chain fatty acid (SCFA) production (Sapp et al., 2022). Zhang et al. (2012) found that a high‐fat diet, in contrast to a regular diet, decreases the abundance of Bacteroidetes and Bifidobacterium in the gut microbiota of rats while increasing the abundance of Firmicutes. The significant enrichment of Firmicutes in the gut microbiota of the WP group strongly supports the aforementioned result. PICRUSt prediction analysis showed that after 35 days of feeding with different foods, the WP group had the highest metabolic abundances among the five groups with different feeding, while the WH group had the lowest. These results indicate that the abundance of specific flora is closely related to the nutrient composition of food, and the intake of different nutrients leads to changes in energy metabolism. In addition, after 35 days of feeding with different foods, the body weight of *C. longicaudatus* in the WH, WA, WP, and WM groups showed a decreasing trend (Figure 7a). We analyzed the correlation between the top 10 genera of the gut microbiota and body weight and found that *Lactobacillus* was significantly positively correlated with body weight (Figure 7b). It has been reported that *Lactobacillus* can prevent and treat obesity by regulating gut microbiota and intestinal permeability, improving insulin resistance, reducing low‐grade systemic inflammation caused by a high‐fat diet, activating brown fat, and regulating the expression of genes related to fat (Cani et al., 2007; Kang & Cai, 2018; Tenorio‐Jiménez et al., 2020).

The gut microbiota exhibits high plasticity, and the establishment of the original microbiota may involve both maternal inheritance and individual autonomous selection (Bruijning et al., 2022; Moeller et al., 2018). However, environmental factors such as dietary patterns, climatic conditions, altitude changes, and human activities are crucial for shaping the evolution of gut microbiota (Gao et al., 2023; López‐Almela I et al., 2021; Zhu et al., 2020). This study investigated the impact of dietary structure regulation on the gut microbiota of *C. longicaudatus* and highlighted the necessity to further explore the relationship between succession of gut microbiota and physiological activities from multiple perspectives including genetic factors and other environmental influences.

## 5 CONCLUSION

The study suggests that dietary structure can significantly impact the diversity and community composition of gut microbiota in *C. longicaudatus*, which primarily inhabits dryland farming areas. The abundance of specific microbiota is closely correlated with the nutrient content of the diet. Gut microbiota plays a pivotal role in regulating nutrient assimilation and metabolic homeostasis in animals. Bacteroidetes exhibit carbohydrate degradation activity. Firmicutes are strongly associated with fat uptake in *C. longicaudatus*. *Lactobacillus* plays a crucial role in modulating fat intake and weight management.

## AUTHOR CONTRIBUTIONS

**Kanglin Cao:** Data curation (lead); formal analysis (lead); investigation (equal); validation (lead); visualization (lead); writing – original draft (lead); writing – review and editing (lead). **Mengfan Tao:** Investigation (equal); resources (equal). **Xinsheng Pu:** Investigation (equal); resources (equal). **Yu Hou:** Investigation (equal); validation (supporting). **Yue Ren:** Data curation (equal); funding acquisition (supporting); supervision (equal). **Wei Liu:** Investigation (equal). **Xin'gen Yang:** Conceptualization (lead); data curation (equal); funding acquisition (lead); supervision (lead); writing – review and editing (equal).

## FUNDING INFORMATION

Shanxi Province Basic Research Program Project, China (202303021221093, 202303021212109), Excellent Doctoral Award of Shanxi Province for Scientific Research (SXBYKY2022123), and the Grant from Shanxi Agricultural University (2023BQ46, 2023BQ47, and ZBXY23B‐14) support for this work.

## CONFLICT OF INTEREST STATEMENT

The authors declare no competing financial interests.

## Data Availability

The 16S rDNA sequencing data supporting this study are available on Dryad at https://doi.org/10.5061/dryad.tx95x6b51.
